# Enhanced Neutrophil Immune Homeostasis Due to Deletion of PHLPP

**DOI:** 10.3389/fimmu.2019.02127

**Published:** 2019-09-06

**Authors:** Taojing Ran, Yao Zhang, Na Diao, Shuo Geng, Keqiang Chen, Christina Lee, Liwu Li

**Affiliations:** ^1^Department of Biological Sciences, Virginia Tech, Blacksburg, VA, United States; ^2^Translational Biology, Medicine, and Health Graduate Program, Virginia Tech, Blacksburg, VA, United States

**Keywords:** inflammation, neutrophils, PHLPP, immune dynamics, colitis

## Abstract

Neutrophils are known to adopt dynamic and distinct functional phenotypes involved in the modulation of inflammation and immune homeostasis. However, inter-cellular signaling mechanisms that govern neutrophil polarization dynamics are not well understood. Employing a novel model of PHLPP deficient mice, we examined how neutrophils deficient in PHLPP may uniquely modulate immune defense and the host response during acute colitis. We found that PHLPP^−/−^ mice were protected from dextran sodium sulfate (DSS)-induced septic colitis characterized by minimal body weight-loss, alleviated colon tissue destruction and reduced clinical symptoms. PHLPP^−/−^ neutrophils have enhanced immune homeostasis as compared to WT neutrophils, reflected in enhanced migratory capacity toward chemoattractants, and reduced expression of inflammatory mediators due to elevated phosphorylation of AKT, STAT1, and ERK. Further, adoptive transfer of PHLPP deficient neutrophils to WT mice is sufficient to potently alleviate the severity of DSS-induced colitis. Our data reveal that PHLPP deficient neutrophils can be uniquely reprogrammed to a state conducive to host inflammation resolution. As a consequence, PHLPP^−/−^ neutrophils can effectively transfer immune homeostasis in mice subjected to acute colitis. Our findings hold significant and novel insights into the mechanisms by which neutrophils can be effectively reprogrammed into a homeostatic state conducive for treating acute injuries such as septic colitis.

## Introduction

Acute colitis caused by infection, injury, and inflammation poses serious health concerns ranging from diarrhea to sepsis. Despite decades of exhaustive research efforts, cellular, and molecular mechanisms underlying the complex pathogenesis of septic colitis remain less well understood. A key stumbling block is the highly dynamic and complex nature of the septic colitis syndrome. With particular relevance, altered functional homeostasis of innate leukocytes such as neutrophils may contribute to compromised host defense as well as inflammation resolution. Proper regulation of neutrophil function is essential for host defense and immune homeostasis. Normal neutrophils in healthy individuals are capable of balanced functions such as chemotaxis and degranulation, as well as secretion of inflammatory mediators (1–4). In sharp contrast, neutrophils from septic patients show compromised chemotaxis coupled with dysregulated expression of inflammatory mediators (5). However, molecular mechanisms underlying the modulation of these neutrophil dynamics in septic colitis are not well studied.

PH domain and Leucine rich repeat Protein Phosphatase (PHLPP) belongs to a novel family of Ser/Thr protein phosphatases that selectively de-phosphorylate both phosphorylated AKT and ERK (6–9). In a less-understood mechanism, PHLPP may also be involved in the modulation of autophagy through its interaction with AKT and other autophagic molecules (10). Through proteomic screening of TOLLIP-interacting proteins, we identified that PHLPP can form a complex with Toll-interacting-protein (TOLLIP), a novel innate immunity adaptor molecule involved in facilitating fusion of the lysosome with either phagosomes or autophagosomes in innate leukocytes (11). TOLLIP deficient neutrophils have reduced phagolysosome fusion and reduced bacterial killing function. At the pathophysiological level, PHLPP deficiency in mice is shown to reduce the severity of acute experimental colitis induced by dextran sodium sulfate (DSS), partly through improved epithelial cell survival, and epithelium integrity in the gut (12). However, the previous study did not address the potential alteration in mucosal immune responses due to PHLPP deficiency as well as the role and regulation of neutrophils related to mucosal immune homeostasis during the course of septic colitis.

Given the important roles that the PHLPP binding partners (TOLLIP, AKT, ERK) play in modulating the functions of neutrophils, PHLPP may also serve as an important regulator that contributes to the dynamic adaptation of neutrophils to inflammatory challenges. PHLPP deficient neutrophils and mice thus provide a unique model system to address the less-understood mechanisms of neutrophil activation during colitis.

In this study, we aimed to test the hypothesis that neutrophils with PHLPP deficiency may adopt a unique activation state conducive for enhanced host immune homeostasis and reduced inflammatory damage in DSS-induced acute septic colitis. We tested the alteration in neutrophil function due to PHLPP deficiency and its contribution to the pathogenesis of septic colitis. We found that PHLPP deficient mice were protected from DSS-induced acute colitis by exhibiting significantly enhanced mucosal immune homeostasis. *In vitro* analyses further confirmed elevated migratory function and reduced expression of inflammatory mediators in PHLPP deficient neutrophils as compared to wild type neutrophils. At the molecular and cellular level, we observed elevated TOLLIP levels and enhanced phagolysosome fusion in addition to elevated STAT1 and AKT activation in PHLPP deficient neutrophils. Further, adoptive transfer of PHLPP deficient neutrophils to WT mice was able to ameliorate symptoms in a septic colitis model. Thus, our data reveal a novel programming dynamics of neutrophils due to PHLPP deficiency that renders enhanced mucosal immune homeostasis.

## Results

### Improved Tissue Homeostasis and Mucosal Immunity in PHLPP^–/–^ Mice

**Figure 1:**
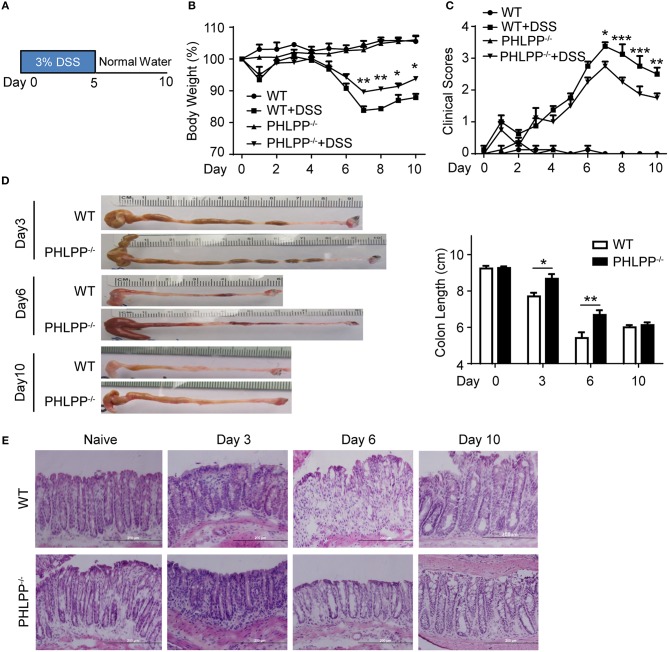
PHLPP deficient mice are protected from DSS-induced acute colitis. **(A)** Schematic of the mice acute colitis model with 3% DSS. **(B)** Weight loss of WT and PHLPP^−/−^ mice treated with regular drinking water or 3% DSS drinking water. Number of mice, *n* = 10 each group. **(C)** Clinical features associated with disease progression demonstrated as clinical score based on daily monitor of weight loss, physical body condition, stool consistency, and rectal bleeding. Number of mice, *n* = 10 each group. **(D)** Length of colons harvested from mice administrated with 3% DSS on day 0, 3, 6, and 10 during the process of experimental colitis. Number of mice, *n* = 5 for day 0, day 3, and day 10; *n* = 12 for day 6. **(E)** Representative images of H&E staining in colon tissues from mice treated with regular drinking water or 3% DSS on day 3, 6, and 10 during the process of experimental colitis. ^*^p < 0.05,^**^p < 0.01, ^***^p < 0.001.

In order to explore the contribution of PHLPP in the context of colitis and how it polarizes neutrophils to modulate colon mucosa immune homeostasis, we used the 3% DSS-induced acute colitis model as described previously (Figure 1A) (13, 14). Mice were sacrificed on day 0, day 3, day 6, and day 10, and pathological observations were documented. We confirmed the previous finding (12) that PHLPP^−/−^ mice were protected from DSS induced colitis with PHLPP^−/−^ mice displaying significantly less body weight loss (Figure 1B) and lower clinical scores as compared to WT mice (Figure 1C). Consistent with the clinical scores, PHLPP^−/−^ mice displayed significantly longer colon lengths (Figure 1D) indicating less colon destruction and effective homeostatic restoration. Further histological H&E staining analysis revealed that PHLPP^−/−^ mice demonstrated less epithelium architectural distortion, lymphocyte infiltration and crypt damage (Figure 1E). Our Ki67 immunofluorescence staining analyses (Supplementary Figure S3) demonstrated that there were more proliferative cells in the colon crypt areas in PHLPP^−/−^ mice as compared to WT mice both at day 3 and day 6 following DSS administration. Together, these analyses all indicated that PHLPP deficiency rendered a protective role during DSS induced colitis.

**Figure 2:**
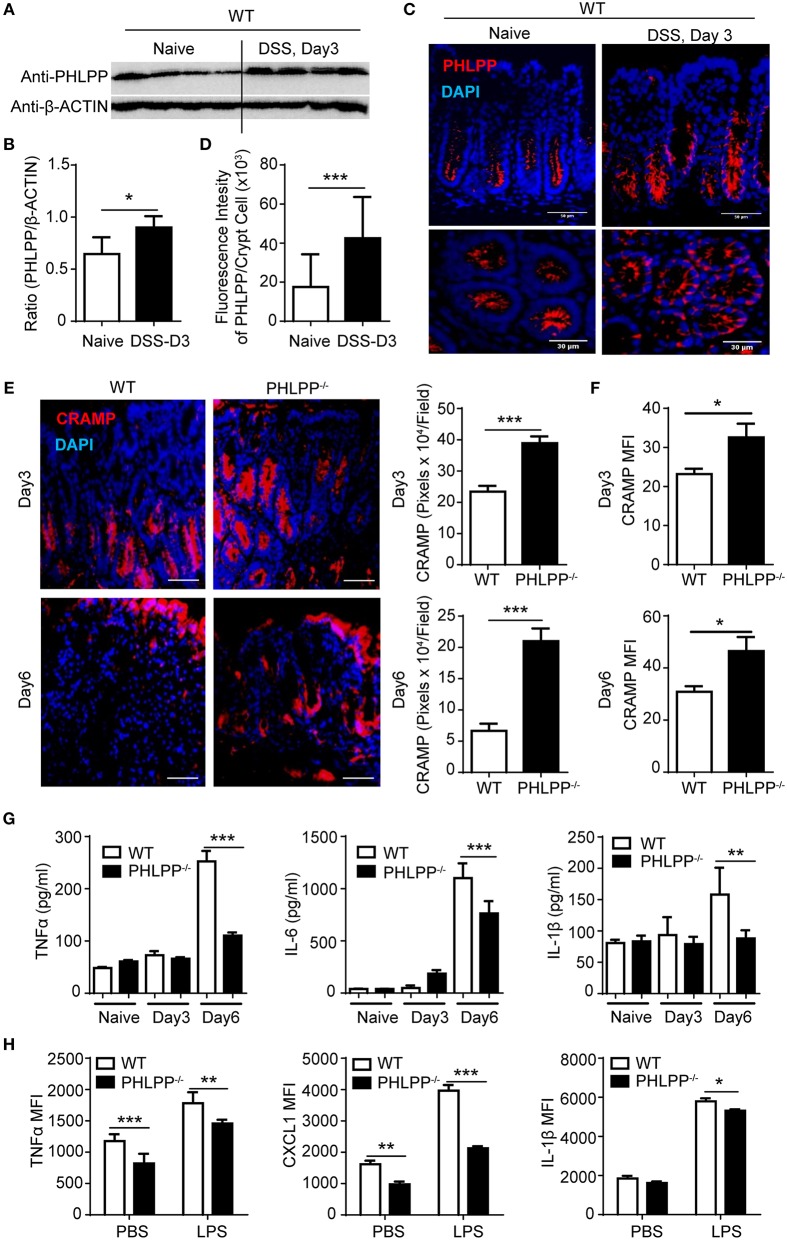
Improved mucosal immune homeostasis in PHLPP deficient mice during acute colitis. **(A)** Immunoblotting analysis of PHLPP expression in colon tissues from naïve WT mice or 3% DSS for 3 days. **(B)** Quantification of immunoblotting analysis of PHLPP protein. *n* = 4 each group. **(C)** Representative images of immunohistological staining of PHLPP in colon tissue of WT mice administrated with regular water or DSS for 3 days. **(D)** Intensity of PHLPP/Crypt was analyzed by image J *n* > 100 each group. **(E)** Representative images of immunohistological staining of CRAMP (left) in colon tissue from WT and PHLPP^−/−^ mice administrated with DSS for 3 or 6 days. Fluorescence intensity of CRAMP/field was analyzed by image J (right). *n* = 9 each group. **(F)** Mean fluorescent intensity of CRAMP in peritoneal neutrophils (LY6G^+^CD11B^+^) harvested from mice administrated with DSS for 3 or 6 days. *n* = 4 each group. **(G)** Cytokine levels in colon tissues collected on Day 0, Day 3, Day 6 during the process of experimental colitis. *n* = 4 each group. **(H)** Cytokine production by neutrophils (LY6G^+^CD11B^+^) were measured by flow cytometry. Purified bone marrow neutrophils were treated with LPS or PBS as control, and Golgi stopper was added for 4 h, then the cells were subjected to cytokine staining. *n* = 6 each group. ^*^p < 0.05, ^**^p < 0.01, and ^***^p < 0.001.

To further confirm the pathological relevance of PHLPP in DSS colitis, we next harvested the colon tissue for Western blot analyses and found that PHLPP expression levels were significantly increased in WT mice treated with DSS for 3 days as compared to controls (Figures 2A,B). Consistent with Western analyses, immunofluorescence staining showed increased tissue levels of PHLPP, especially in the colon crypt area during the onset of DSS-induced acute colitis (Figures 2C,D). We further purified colon neutrophils from WT mice challenged with DSS for 3 days and observed the expression of PHLPP in purified colon neutrophils (Supplementary Figure S4).

Despite the previously observed improved colitis outcome, no existing study is currently available to address the alteration in mucosal immune homeostasis due to PHLPP deficiency. Thus, we examined the tissue levels of antimicrobial peptides such as cathelin-related antimicrobial peptide (CRAMP), a key player of mucosal defense generated by neutrophils within the gastrointestinal tract (15). CRAMP is also a potent ligand for neutrophil chemotaxis through the FPR2 receptor. We observed significantly elevated levels of CRAMP within colon tissues of PHLPP^−/−^ mice post DSS administration as shown by immunohistochemistry analyses (Figure 2E). We further confirmed that PHLPP^−/−^ neutrophils expressed significantly higher levels of CRAMP (Figure 2F). Next, we measured the levels of inflammatory mediators such as TNFα, IL-1β, and IL-6 in colon tissues from WT and PHLPP deficient mice challenged with DSS at various time points. As shown in Figure 2G, the levels of TNFα, IL-1β, and IL-6 in colon tissues of WT mice remained high at d 6 post DSS challenge, correlated with severe tissue damage and worse colitis outcome as observed above. In sharp contrast, the levels of TNFα, IL-1β, and IL-6 in colon tissues of PHLPP deficient mice were significantly lower, consistent with improved tissue immune homeostasis due to PHLPP deficiency. We also examined the *in vitro* expression of selected inflammatory cytokines. Purified neutrophils were stimulated with either LPS or PBS control in the presence of Golgi stop solution, followed by subsequent FACS analyses. Consistent with *in vivo* observations, PHLPP^−/−^ neutrophils exhibited significantly reduced production of TNFα, IL-1β, and CXCL1 in response to LPS stimulation as compared to WT neutrophils (Figure 2H). Together, our data demonstrate PHLPP deficiency improves effective mucosal innate immune homeostasis in DSS-treated mice.

### Enhanced Neutrophil Migratory Functions *in vivo* Due to PHLPP Deficiency

**Figure 3:**
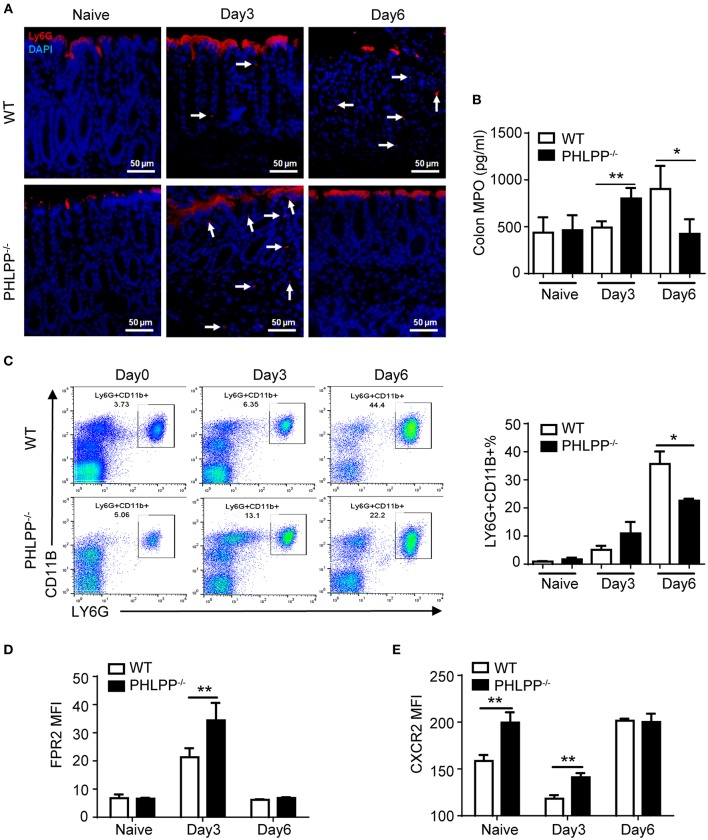
Enhanced neutrophil activation due to PHLPP deficiency. **(A)** Representative images of immunohistological staining of neutrophils (LY6G+) in colon tissues of DSS-treated WT and PHLPP^−/−^ mice on day 0, 3, and 6 during the process of experimental colitis. **(B)** Colon tissues were collected on day 0, 3, and 6 cultured overnight. Supernatant was collected for detection of MPO by ELISA. **(C)** Flow cytometry analysis of peripheral neutrophils (gated on CD11B+ LY6G+) harvested on day 0, 3, and 6 during the process of experimental colitis. **(D)** FPR2 expression on peripheral neutrophils (gated on LY6G+ CD11B+) from WT and PHLPP^−/−^ mice on day 0, 3, and 6 post DSS challenges via flow cytometry. **(E)** CXCR2 expression on peripheral neutrophils (gated on LY6G^+^CD11B^+^) from DSS-treated mice on day 0, 3, and 6 post DSS challenges via flow cytometry. For mouse number in each group, *n* ≥ 4. ^*^p < 0.05, ^**^p < 0.01.

Given our observation of elevated mucosal neutrophil-secreted peptide CRAMP and reduced inflammatory cytokines in PHLPP deficient mice, we next specifically examined the alterations in neutrophil functions due to PHLPP deficiency. We first examined the levels of neutrophils in colon tissue by immunofluorescence staining of LY6G and secretion of MPO (myeloperoxidase, a key marker of tissue neutrophils). Compared to WT mice, PHLPP^−/−^ mice showed more colonic MPO expression after 3 Days DSS administration and this trend reversed on Day 6 (Figures 3A,B). The higher initial influx of neutrophils coincided with more effective mucosal immune defense and the later regression of neutrophils coincided with better immune homeostasis at the later stage of DSS challenge in PHLPP deficient mice. In contrast, neutrophils in WT mice exhibited a slower recruitment response and lasted longer in DSS-challenged mice colon tissues, corresponding to a compromised earlier phase of host defense and a severe late phase of tissue inflammation/damage. Moreover, much higher percentages of neutrophils in the peripheral blood were also observed in WT mice than in PHLPP deficient mice on Day 6 (Figure 3C), indicating prolonged and severe inflammation in WT mice compared with PHLPP deficient mice.

At the molecular level, we examined the expressions of chemoattractant receptors (FPR2 and CXCR2) on the peripheral neutrophils via flow cytometry (16). We first examined the levels of FPR2, the receptor for CRAMP. This is based on our observation that the levels of CRAMP were increased in PHLPP deficient neutrophils, likely responsible for the elevated recruitment of neutrophils to the damaged colon tissues. Compared to WT mice, PHLPP^−/−^ peritoneal neutrophils expressed significantly higher levels of FPR2 on Day 3 post DSS challenge as compared to WT neutrophils (Figure 3D). In contrast, the levels of FPR2 on PHLPP deficient neutrophils leveled off to the levels similar to WT neutrophils on Day 6 post DSS challenge (Figure 3D). CXCR2 levels in WT neutrophils are known to be acutely reduced following inflammatory challenges (17). Compared to WT mice, PHLPP^−/−^ neutrophils expressed significant higher levels of CXCR2 on day 0 and day 3 post DSS challenge (Figure 3E). The levels of CXCR2 on PHLPP deficient neutrophils also returned to similar levels as compared to WT neutrophils on day 6 post DSS challenge. These data complemented our above-mentioned phenotypic observation that, during the early phase of colon damage by DSS, PHLPP^−/−^ neutrophils were more effectively recruited to the colon tissue, defending against bacterial infiltration. On the other hand, WT neutrophils exhibited less efficient infiltration to the colon tissues during the early phase of colon damage, and were retained longer in colon tissues contributing to more severe tissue damage.

### Enhanced Neutrophil Migratory Functions *in vitro* Due to PHLPP Deficiency

**Figure 4:**
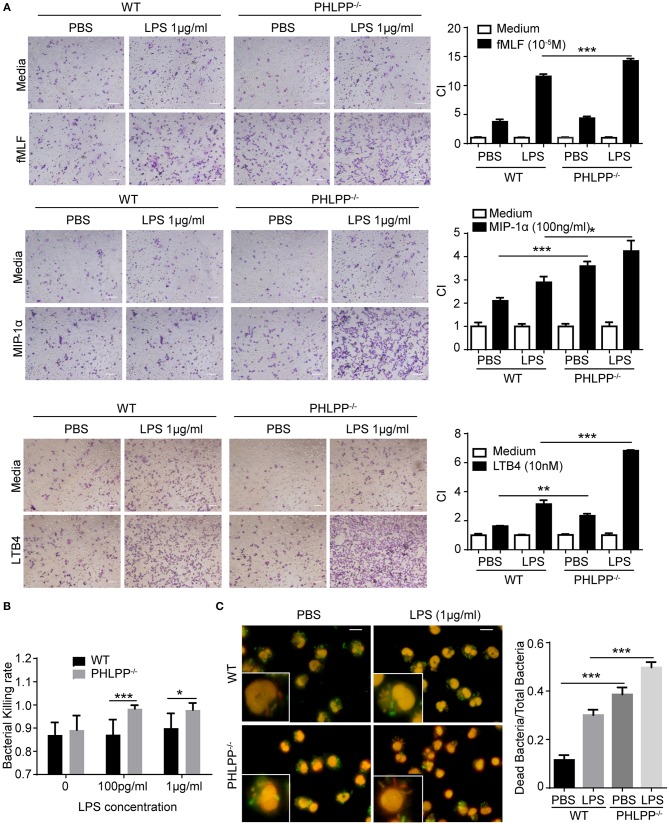
Enhanced neutrophil migratory function as well as elevated bacterial killing activity *in vitro* due to due to PHLPP deficiency. **(A)** Chemotaxis of neutrophils from bone marrow to fMLF, MIP-1α, or LTB4. Bone marrow neutrophils were primed with LPS (1 μg/ml) or PBS as control overnight, then subjected to the chemotaxis assay. Macroscopic observations of chemotaxis chamber were grouped on the left, and quantification of migrated neutrophils was shown in bar graph on the right. *n* = 3 each group. Scale bar = 50 μm. ^*^p < 0.05, ^**^p < 0.01, and ^***^p < 0.001. **(B)** Bacterial killing assay through plating. *n* = 6 each group. **(C)** Intracellular assay of live and dead bacteria through differential staining. Neutrophils were infected with *E.coli*, then exposed to the live/dead staining kit with propidium iodide and SYTO9. Live bacteria appeared green, dead bacteria appeared red and nuclei appear pink (left). Bacterial killing ability was analyzed by dead bacterial/total bacterial x100% (right). *n* = 80 each group. ^***^p < 0.001.

Based on the above *in vivo* observation, we next studied the migratory functions of PHLPP deficient neutrophils *in vitro*. In terms of the migratory potential, we tested several well-established neutrophil chemoattractants including formyl-methionyl-leucyl phenylalanine (fMLF), leukotriene B4 (LTB4), and macrophage inflammatory protein-1α (MIP-1α). We cultured neutrophils isolated from the bone marrow of WT and PHLPP^−/−^ mice with either PBS or high dose LPS (1 μg/ml) stimulation overnight. Cultured neutrophils were then tested for migratory potential toward above-mentioned chemoattractants via the chemotaxis chamber assay. We observed significantly enhanced migration ability toward all chemoattractants tested in PHLPP^−/−^ neutrophils as compared with WT neutrophils (Figure 4A, Supplementary Figure S5). To supplement our migratory assay with the conventional migratory chamber, we further performed migration assays using microfluidic chambers coupled with live cell imaging analyses for cellular migration. We observed a robust and dynamic migration of PHLPP deficient neutrophils toward LTB4 which peaked around 15 h following the start of the assay (Supplementary Figure S5). Together, our study reveals that PHLPP deficiency leads to elevated signaling processes that contribute to enhanced migratory potential of neutrophils.

We further examined the bacterial killing functions of PHLPP deficient neutrophils. To test this, we incubated *E. Coli* bacteria with WT and PHLPP^−/−^ neutrophils primed with or without LPS for 1 h and examined the bacteria-killing activity through cell lysis and plating. We observed that PHLPP^−/−^ neutrophils demonstrated significantly elevated bacterial killing activity as compared to WT neutrophils (Figure 4B). We also used an independent Live/Dead staining assay to directly measure intra-cellular bacteria killing within WT and PHLPP^−/−^ neutrophils. We found that compared to WT neutrophils, the ratio of dead intra-cellular bacteria in PHLPP^−/−^ neutrophils was much higher, further supporting a more efficient bacterial killing function of PHLPP^−/−^ neutrophils (Figure 4C).

### PHLPP Deficiency Elevates TOLLIP-Mediated Lysosome Fusion With Phagosome

**Figure 5:**
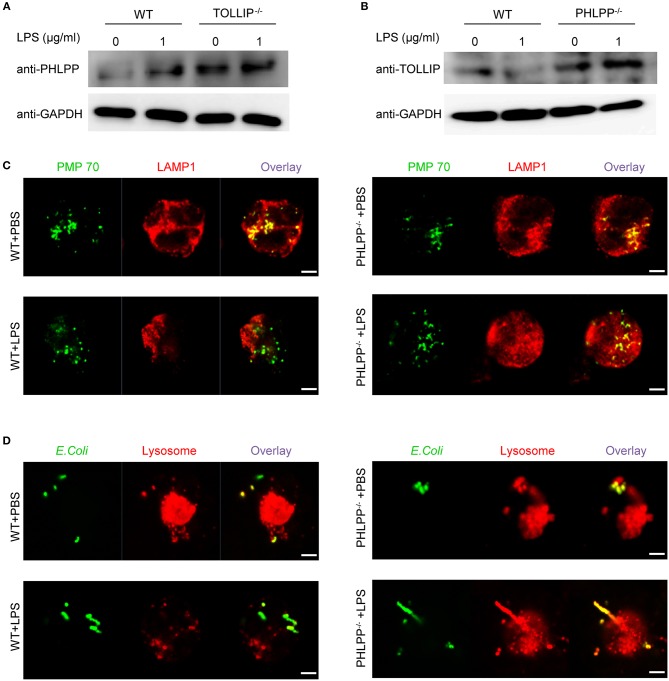
PHLPP antagonizes the function of TOLLIP and modulates lysosome fusion. **(A)** Immunoblotting of purified neutrophils from bone marrow of WT or TOLLIP^−/−^ mice stimulated with LPS (1 μg/ml) or PBS as control overnight. **(B)** Immunoblotting of purified neutrophils from bone marrow of WT or PHLPP^−/−^ mice stimulated with LPS (1 μg/ml) or PBS as control overnight. **(C)** WT or PHLPP^−/−^ neutrophils were treated with PBS or LPS overnight, followed by staining with anti-PMP70 and anti-LAMP1 antibodies. The fusion of peroxisomes with lysosomes was visualized via confocal microscopy. **(D)** WT or PHLPP^−/−^ neutrophils were co-cultured with FITC-labeled *E.coli* for 1 h, and lysosomes were stained with LysoTracker™ Red DND-99. The delivery of *E.coli* into lysosomes was visualized via confocal microscopy. Scale bar = 2 μm.

We next aimed to better understand the underlying molecular and cellular mechanisms responsible for the enhanced neutrophil homeostasis due to PHLPP deficiency. The observed functional phenotypes of PHLPP deficient neutrophils were opposite from those observed in our previous report of TOLLIP deficient neutrophils (14). In contrast to PHLPP, we previously reported that TOLLIP is required for the fusion of the lysosome with the phagosome and critical for facilitating neutrophil homeostasis, while TOLLIP deficient neutrophils have compromised lysosome fusion and enhanced expression of inflammatory mediators (11, 14). Thus, we tested the potential interaction between TOLLIP and PHLPP in neutrophils by comparing the cellular levels of PHLPP in WT and TOLLIP deficient neutrophils by Western blot. TOLLIP deficient neutorphils had significantly higher levels of PHLPP protein as compared to WT neutrophils (Figure 5A). On the other hand, PHLPP deficient neutrophils also showed elevated levels of TOLLIP (Figure 5B), thus suggesting a mutually competitive inhibition between TOLLIP and PHLPP.

Since TOLLIP is a critical regulator of lysosome fusion, we tested whether PHLPP deficiency may enhance lysosome fusion in neutrophils. Indeed, as compared to WT neutrophils, we observed enhanced fusion of lysosome with phagocytosed bacteria, as well as with the peroxisome in PHLPP deficient neutrophils treated with LPS (Figures 5C,D). The enhanced lysosomal fusion in PHLPP deficient neutrophils supports the previously observed enhanced neutrophil homeostasis due to PHLPP deficiency.

**Figure 6:**
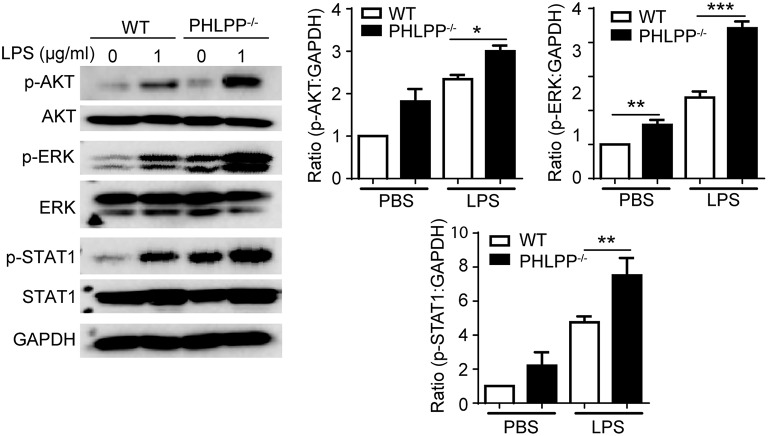
PHLPP deficiency elevates the activities of AKT, ERK and STAT1. Immunoblotting of purified neutrophils from bone marrow stimulated with LPS (1 μg/ml) or PBS as control overnight. Right panels are blot quanifications (*n* = 3) ^*^p < 0.05, ^**^p < 0.01, and ^***^p < 0.001.

In terms of molecular mechanisms, we further examined the activation status of STAT1 and ERK. STAT1 has been identified as a critical mediator of the innate leukocyte response to infection, and the loss of STAT1 is correlated with compromised bactericidal ability (18). ERK and AKT are both critically involved in neutrophil migration as well as enhancing neutrophil homeostasis through suppressing the expression of pro-inflammatory mediators (19–21). Using Western Blot analysis, we examined the phosphorylation of STAT1, ERK, and AKT, and observed significantly enhanced levels of p-STAT1, p-ERK, and p-AKT in PHLPP deficient neutrophils (Figure 6), consistent with their enhanced homeostasis. PHLPP^−/−^ neutrophils also exhibited elevated STAT5 levels. The enhanced activities of ERK and lysosome fusion can not only contribute to enhanced bacterial killing and neutrophil chemotaxis, but are also associated with more effective resolution of cellular stress and inflammatory activation (22). Indeed, as shown in Figure 2G, we observed significantly reduced levels of inflammatory mediators such as TNFα from PHLPP^−/−^ mice challenged with DSS as compared to WT mice. Collectively, our data reveal that PHLPP deficiency contributes to more effective neutrophil homeostasis, potentially through enhanced activities of AKT/STAT1 and enhanced lysosome fusion.

### Adoptive Transfer of PHLPP Deficient Neutrophils Is Sufficient to Alleviate the Severity of DSS Induced Septic Colitis

**Figure 7:**
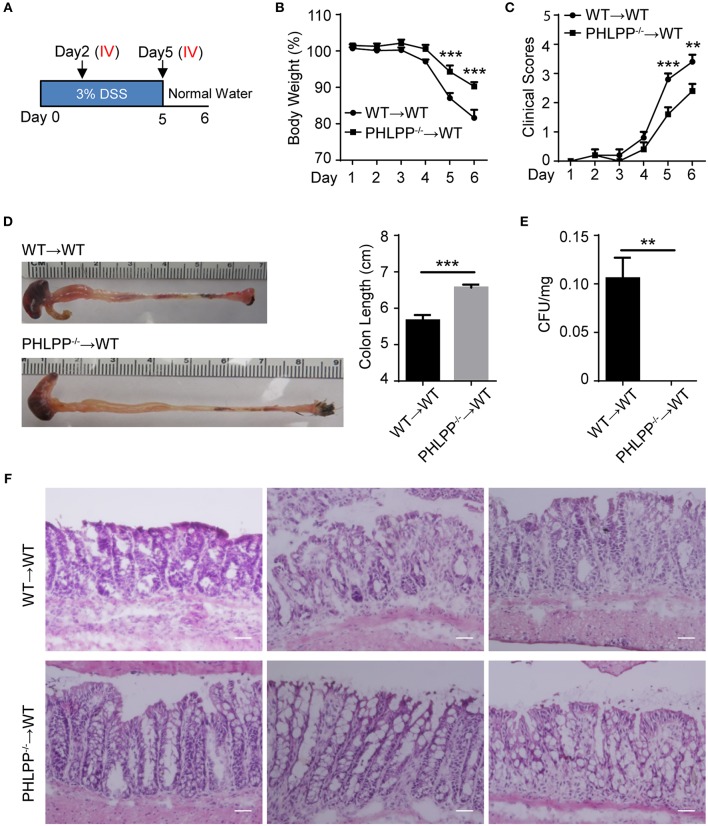
Adoptive transfer of PHLPP deficient neutrophils is sufficient to render protection against DSS-induced colitis. **(A)** Schematic drawing of the experimental design for the WT and PHLPP^−/−^ neutrophil adoptive transfer in the DSS-induced acute colitis model. **(B)** Weight loss of WT or PHLPP^−/−^ neutrophil recipients. *n* = 5 each group. **(C)** Clinical scores based on weight loss, physical body condition, stool consistency, and rectal bleeding. *n* = 5 each group. **(D)** Length of colon harvested from WT or PHLPP^−/−^ neutrophil recipients. *n* = 5 each group. **(E)** Bacterial counts of spleen harvested from WT or PHLPP^−/−^ neutrophil recipients. *n* = 5 each group. **(F)** H&E staining of colon tissues from WT or PHLPP^−/−^ neutrophil recipients. Scale bar = 50 μm. ^**^p < 0.01, ^***^p < 0.001.

To test our hypothesis that PHLPP deficiency in neutrophils would be sufficient to render a protective effect on colitis, we performed an adoptive transfer study. Either WT or PHLPP^−/−^ neutrophils were *i.v*. transfused to recipient WT mice on day 2 and day 5 during DSS challenge, and sacrificed on day 6 (Figure 7A). WT mice transfused with PHLPP^−/−^ neutrophils exhibited significantly less body weight loss, reduced clinical disease scores, and reduced colon shortening as compared to mice transfused with WT neutrophils (Figures 7B–D). In order to evaluate the severity of septic colitis, we next digested the spleen for bacterial count, and observed significantly reduced bacteria from mice transfused with PHLPP^−/−^ neutrophils as compared to mice transfused with WT neutrophils (Figure 7E). H&E staining also revealed that colon tissues from mice transfused with PHLPP^−/−^ neutrophils had improved overall gut integrity (Figure 7F). Together, these results suggest that PHLPP deficiency is sufficient to reduce the severity of DSS-induced septic colitis in mice and improve clinical outcomes.

## Discussion

In this study, we reveal a novel protective role of re-programmed neutrophils contributing to enhanced mucosal immune homeostasis during chemically-induced septic colitis due to PHLPP deficiency. Our conclusion is supported collectively by the evidence from both *in vitro* and *in vivo* experiments. PHLPP^−/−^ neutrophils adopt an unique homeostasis state with enhanced chemotaxis and reduced expression of pro-inflammatory mediators as compared to WT neutrophils. Mechanistically, PHLPP deficiency leads to increased AKT and STAT1 activation, as well as enhanced phago-lysosomal fusion, conducive to enhanced neutrophil homeostasis. *In vivo* analyses reveal that PHLPP^−/−^ mice are protected from DSS-induced septic colitis, with reduced tissue inflammation and effective homeostasis. Our adoptive transfer study indicates that reprogrammed neutrophils due to PHLPP deficiency are sufficient to transfer beneficial protection against DSS-induced septic colitis.

Besides confirming the previously identified protective effect of whole body PHLPP deletion in mice toward DSS-induced colitis, our data reveal novel roles of re-programmed neutrophils due to PHLPP deficiency. Emerging studies suggest highly complex neutrophil programming dynamics and their pivotal roles in balancing inflammation and homeostasis. Controversies still exist with regard to conflicting dual roles that neutrophils may play in either causing detrimental exacerbation of inflammation or facilitating beneficial inflammation resolution (23). Our previous studies demonstrate that innate leukocytes, especially monocytes and macrophages, can be dynamically programmed into distinct memory states (23–26). As a consequence, the distinct innate “memory” immune cells may differentially affect the pathogenesis of acute and chronic inflammatory diseases (14, 27, 28). Our current study extends these previous studies and reveals novel “programming” of neutrophils due to PHLPP deficiency, which is conducive to tissue homeostasis. Our data show that, when challenged with DSS, neutrophils from PHLPP^−/−^ mice responded much more quickly to the colon as compared to the neutrophils from WT mice during the acute phase of DSS-induced colon damage (day 3 post DSS). This is also reflected in higher levels of MPO in WT mice as compared to PHLPP^−/−^ mice 3 day post DSS challenge. In contrast, WT mice experience a slower build-up of neutrophils at the early phase of DSS-induced colon damage, and more persistent accumulation of neutrophils at d 6 post DSS, coinciding with higher levels of inflammatory mediators in WT mice and elevated tissue damage as compared to PHLPP deficient mice at the later phase of colitis. In contrast, DSS-challenged mice as well as harvested neutrophils from PHLPP deficient mice have significantly reduced expression of pro-inflammatory mediators, consistent with enhanced mucosal immune homeostasis due to PHLPP deficiency. Despite a previous report that demonstrated a reduction in the mucosal epithelial levels of PHLPP following DSS challenge (12), our data has robustly shown an increase in the levels of PHLPP in neutrophils from WT mice challenged with DSS, corresponding to increased tissue inflammation and damage in WT mice. Our data collectively reveal that PHLPP deficiency can program neutrophils into a homeostatic state conducive for inflammation resolution.

Our study also clarifies novel molecular and cellular mechanisms that underlie the homeostatic programming of neutrophils. Previous studies indicated that several key signaling pathways such as AKT and ERK1/2 activation (29, 30) are involved in neutrophil ROS production, degranulation, and chemotaxis. PI3K/AKT and ERK signaling pathways also regulate endosome-lysosome trafficking and the induction of neutrophil autophagy which is critical for inflammatory resolution and immune cell survival (31). PHLPP was identified as a selective phosphatase in epithelial cells as well as in cancer cells that dephosphorylates AKT and ERK1/2 thus suppressing the PI3K/AKT and Ras/ERK signaling pathway (32, 33). To our knowledge, our current study provides the first of its kind link of PHLPP to the regulation of AKT/ERK activities in neutrophils. In addition, our data reveal that PHLPP is also involved in the modulation of STAT1, which is critical for the activation of innate leukocytes in response to pathogen infections (18, 34, 35). PHLPP deficient neutrophils have significantly increased levels of STAT1 activation and increased bacteria-cidal activities. Our study further reveals the role of PHLPP during the sub-cellular process of phagolysosome fusion. We observed that PHLPP deficient neutrophils have enhanced lysosome fusion with the phagosome as well as with the peroxisome. Collectively, our data suggest key subcellular and molecular bases involved in PHLPP-mediated neutrophil homeostasis.

There are rising interests from both basic and translational studies to harness the therapeutic potential of *ex vivo* re-programmed leukocytes in treating human diseases, exemplified by the recent success of cell-based cancer therapies using engineered T cells (36). However, there has been limited studies with innate leukocytes such as neutrophils, although emerging studies suggest the existence of distinct “memory” neutrophil states associated with pathogenesis and/or resolution of inflammatory diseases (23). Our adoptive transfer data with PHLPP neutrophils suggest a proof-of-principle that re-programmed neutrophils may hold therapeutic potential in treating septic colitis.

Taken together, our data reveal that the reprogramming of neutrophils due to PHLPP deficiency may hold novel potential in enhancing mucosal immune homeostasis. Our mechanistic studies suggest that PHLPP is involved in a competitive circuit involved in modulating neutrophil homeostasis. Future studies that further examine the interaction of PHLPP with its neighboring signaling partners are needed in order to better understand the neutrophil programming dynamics during their adaptation to distinct activation states. The effects of reprogrammed neutrophils due to PHLPP deficiency on other immune cells such as adaptive T cells will also require future extensive studies, in order to better evaluate and harness the therapeutic potential in treating septic colitis.

## Methods

### Experimental Animals

PHLPP^−/−^ mice were purchased from the Jackson Laboratory. C57BL/6 and PHLPP^−/−^ mice were bred at the animal facility at Virginia Tech. All animal studies were reviewed and approved by the Institute for Animal Care and Use Committee (IACUC) at Virginia Tech. Mice used in the experiment were 6–8 weeks of age and 20–30 g of weight. Mice were properly anesthetized and euthanized in accordance with the approved animal protocol before tissue harvest and analyses.

### Reagents

LPS (*Escherichia coli* 0111:B4) was from Sigma-Aldrich. Dextran sulfate sodium (DSS, MW 47,000; MP Biomedicals) was purchased from MP Biomedicals. Antibodies against phospho-AKT473, phospho-ERK1/2, phosphor-STAT1, AKT, ERK1/2, STAT1, GAPDH were purchased from Cell Signaling Technology, PHLPP was from EMD Millipore, β-ACTIN was from Santa Cruz. FITC conjugated anti-mouse LY6G antibody, Alexa Fluor 647 conjugated anti-mouse CD182 (CXCR2) Antibody, APC/Cy7 and PE conjugated anti-mouse/human CD11B antibody were from Biolegend. ELISA Kit for TNFα, IL-1β, and IL-6 were from eBioscience, respectively. MPO ELISA Kit from R&D system. leukotriene B4 (LTB4), N-Formyl-Met-Leu-Phe (fMLF), and percoll were purchased from Sigma-Aldrich. EasySep™ Mouse Neutrophil Enrichment Kit was purchased from Stemcell Technologies. MIP-1α, fibronectin, and G-CSF was from Peprotech. Anti-PMP70 was from ThermoFisher Scientific. Anti-LAMP was from Abcam.

### DSS-Induced Colitis Model

WT and PHLPP^−/−^ mice were given 3.0% (w/v) DSS in drinking water continuously for 5 days, followed with regular water for an additional 5 days. Control group were given regular drinking water during the whole experiment (Figure 1A).

### Adoptive Transfer of Neutrophils

WT mice aged 6 weeks were divided into two recipient groups transfused with either WT neutrophils or PHLPP^−/−^ neutrophils. All recipients were given 3.0% (w/v) DSS in drinking water continuously for 5 days followed with regular water for 1 day. Purified bone marrow neutrophils were re-suspended in PBS to the concentration of 25 × 10^6^ cells/ml. 5 × 10^6^ cells were adoptively transfused each time to recipient mice by tail intravenous injection on Day 2 and Day 5 during the DSS colitis model. Mice were sacrificed on Day 6 (Figure 7A).

### Disease Scoring Criteria

Clinical scores were assessed covering the perimeters shown in Supplementary Figure S1. Characteristics of acute colitis were monitored daily by documenting weight loss, physical body condition, stool consistency, and rectal bleeding.

### H and E Staining and Immunofluorescence

Histology analyses were performed on the 5 μm frozen, Optimal-Cutting-Temperature compound (OCT) embedded and sectioned slides, stained with hematoxylin and eosin. Immunofluorescence analyses were also performed on freshly frozen, OCT embedded colon tissues. Samples were sectioned (10 μm) and subsequently stained with anti-mouse primary antibody (anti-CRAMP 1:1000, anti-LY6G 1:1000, anti-Ki67 1:1000) followed by a biotinylated anti-IgG secondary antibody and Streptavidin- PE or fluorescein isothiocyanate. DAPI was used to stain the nucleus. Multiple viewing fields from each slide were captured using a fluorescence microscope. Pixel values reflecting the fluorescence intensities of each image were quantified with NIH ImageJ software.

### Colon Tissue Culture and Pro-inflammatory Mediator Assessments

The colon tissue was washed with PBS, split longitudinally and the distal 3 cm colon tissues were cut into 1–2 cm pieces. All the biopsy specimens were then transferred into 24-well tissue culture plate covered with RPMI 1640 medium supplemented with 1% FBS and 1% penicillin/streptomycin and incubated overnight. The supernatants of the colon tissue culture were collected for further ELISA assay.

### Neutrophil Purification and Culture

Neutrophils were isolated from WT and PHLPP^−/−^ mice bone marrow. Briefly, bone marrow cells re-suspended in RPMI 1640 were layered over a percoll gradient (55,65, and 82%) and centrifuged at 1,100 g for 30 min. The dense band at 65/82% interface was collected as the neutrophil fraction. The purities of neutrophils were >90% as assessed by flow cytometry analysis (Supplementary Figure S2). Isolated neutrophils were cultured with RPMI completed medium (10% FBS, 1 %penicillin/streptomycin, 1 % glutamine) supplemented with 100 ng/ml G-CSF and stimulated with or without LPS (1 μg/ml) at 37 °C in 5% CO_2_ overnight. The cells were harvested next day for further analysis. Neutrophils for adoptive transfer purpose were purified by EasyStep mouse neutrophil enrichment kit according to the instruction from the manufacturer. For isolation of neutrophils from colon, the colon tissues were removed from mice fed with DSS for 3 days, and washed with PBS, cutted into pieces (~0.5 cm length), and then dissociated with Lamina Propria Dissocation Kit from MACS according to the manufacturer's instructions. Total single cell suspension was carefully loaded on percoll gradient (65 and 82%), centrifuged and collected as described above. Purified neutrophils were subjected to immunoblotting with anti-PHLPP antibody.

### ELISA of Cytokines

Periphal blood was collected into EDTA-coated tubes. After centrifugation, plasma was collected and stored in −80°C for later analysis. TNFα, IL-1β, and IL-6 levels in the supernatants of colon tissue culture were analyzed by enzyme-linked immunosorbent assay (ELISA) using ELISA Kit from eBioscience according to the manufacturer's instructions. Myeloperoxidase (MPO) levels of colon culture supernatants and plasma were assessed using mouse MPO ELISA Kit from R&D system.

### Chemotaxis Assays

Chemotaxis of neutrophils was analyzed by 48-well micro-chambers with 5 μm pore-size polycarbonate filters. Purified neutrophils were pre-treated overnight as described above, and re-suspended in the RPMI 1640 medium (1% BSA, 1% penicillin/streptomycin, 1% L-glutamine). A final volume of 50 μl of cells was loaded into the upper chamber and the lower chambers were filled with 30 μl chemoattractants (fMLF 10^−5^ M, MIP-1α 100 ng/ml, LTB4 10 nM or 100 nM). The chambers were incubated at 37°C in 5% CO_2_ for 2 h. The migrated neutrophils were counted under a microscope and chemotaxis ability was demonstrated as the chemotaxis index (CI), representing the fold increase in the number of migrated cells in response to chemo-attractants over spontaneous cell migration (toward control medium).

To measure neutrophil migration in real time, purified neutrophils were resupended at the concentration of 12 million cells per ml in RPMI 1640 completed medium supplemented with 75 μg/ml fibronectin. Neutrophils were primed with LPS (1 μg/ml) 2 h before loading to microfluid device. The chemoattractant LTB4 (100 nM) was added to the device, and RPMI 1640 was used as control. The migration events were monitored and captured with Nikon Eclipse Ti microscope for 24 h. The images were analyzed with Image J and neutrophils were counted at all time points.

### Immunoblotting

Colon epithelium cells and overnight cultured neutrophils were harvested in 1X SDS lysis buffer containing protease inhibitor cocktail. Protein bands were separated on SDS-PAGE followed by transfer to an Immuno-Blot PVDF membrane. Western blot analyses were performed with the specified antibodies as previously described (27). The PHLPP, p-AKT, p-ERK1/2, p-STAT1, AKT, ERK, STAT1, STAT5, TOLLIP, and GAPDH antibodies were used as 1:1000 dilution. For blot quantification, band intensities were quantified by Image J and relative ratio of WT PBS group was set as 1. The quantification was based on biological triplicates.

### Bacterial Killing Assay

Purified neutrophils were cultured overnight as mentioned above, then adjusted the multiplicity of infection (MOI bacteria/neutrophil) to 5/1. The mixture of cells and bacterial were incubated at 37°C in 5% CO_2_ for 1 h. 20 μl mixed solution of neutrophil/bacteria was used to spread on LB gel covered plate. Plates were incubated at 37°C in 5% CO_2_ overnight. Bacteria colonies were counted next day. The bacterial killing rate was calculated as (Total bacteria—Counted bacteria)/Total bacteria.

### Flow Cytometry

Single cell suspensions were stained with fluorescently conjugated antibodies (BioLegend) in the presence of Fc block on ice for 20 min. After washing, the cells were resuspended in flow buffer (1xHBSS/2% FBS), and subjected to flow analysis. Intracellular cytokine staining was performed using Fixation/Permeabilization Solution Kit with BD GolgiStop™ from BD Biosciences. Samples were analyzed with a FACSCanto II (BD Biosciences). FACS plots shown were analyzed with FlowJo (Ashland, OR).

### Confocal Microscopy

WT and PHLPP^−/−^ neutrophils were treated with PBS or LPS overnight. To determine lysosome-peroxisome fusion, the cells were fixed with 4% PFA, deposited on slides through cytospin, and permeabilized with 0.2% Triton X-100. The cells were blocked and stained with primary rabbit anti-mouse PMP70 antibody at room temperature, followed by staining with Alexa Fluor 488-goat anti-rabbit secondary antibody. After extensive washing with PBS, the cells were then stained with Cy3-anti- LAMP1 antibody. To determine the fusion of intracellular bacteria with lysosome, the neutrophils were co-cultured with FITC-labeled *E.coli* for 1 h, and LysoTracker™ Red DND-99 (75 nM) was added to the co-cultures. Then, the extracellular bacteria were removed by extensive washing with PBS, and the cells were fixed with 4% PFA and deposited on slides via cytospin. The samples were observed under a confocal microscope.

To label *E.coli*, freshly cultured bacterial cells were re-suspended in 1 ml of 0.1 M sodium bicarbonate buffer at concentration of 1 × 10^9^ bacteria/ml. 0.2 μl of FITC solution (10 mg/ml in DMSO) was added into cells suspension, then incubated in the dark with end-over-end rotation for 30 min at room temperature. The cells were washed and resuspened in PBS. After measurement of concentration, bacteria was mixed with neutrophils at MOI of 5.

### Statistical Analysis

Data were represented as mean ± standard error of mean (S.E.M.) unless otherwise indicated. Graphs and statistical analysis were conducted via GraphPad PRISM software. Student *t-*test was used for parametric analyses between 2 groups. Complex data sets were analyzed by one way analysis of variance (ANOVA) and followed by either Tukey Kramer HSD or Newman-Keuls method. *P* < 0.05 was considered statistically significant.

## Ethics Statement

This study was carried out in accordance with the recommendations of the Institutional Animal Care and Usage Committee (IACUC) of Virginia Tech. The protocol was approved by the Institutional Animal Care and Usage Committee (IACUC) of Virginia Tech.

## Author Contributions

TR, YZ, ND, SG, CL, and KC designed and performed the experiments and analyzed the data. TR, YZ, and LL wrote the manuscript. LL conceived the study, designed the experiments, and provided conceptual guidance.

### Conflict of Interest Statement

The authors declare that the research was conducted in the absence of any commercial or financial relationships that could be construed as a potential conflict of interest.

## References

[1] Mayadas TN Cullere X Lowell CA. The multifaceted functions of neutrophils. Ann Rev Pathol. (2014) 9:181–218. 10.1146/annurev-pathol-020712-164023 24050624 PMC4277181

[2] Meng W Paunel-Gorgulu A Flohe S Hoffmann A Witte I MacKenzie C. Depletion of neutrophil extracellular traps *in vivo* results in hypersusceptibility to polymicrobial sepsis in mice. Crit Care. (2012) 16:R137. 10.1186/cc11442 22835277 PMC3580722

[3] Arraes SM Freitas MS da Silva SV de Paula Neto HA Alves-Filho JC Auxiliadora Martins M. Impaired neutrophil chemotaxis in sepsis associates with GRK expression and inhibition of actin assembly and tyrosine phosphorylation. Blood. (2006) 108:2906–13. 10.1182/blood-2006-05-024638 16849637

[4] Torres-Duenas D Benjamim CF Ferreira SH Cunha FQ. Failure of neutrophil migration to infectious focus and cardiovascular changes on sepsis in rats: effects of the inhibition of nitric oxide production, removal of infectious focus, and antimicrobial treatment. Shock. (2006) 25:267–76. 10.1097/01.shk.0000208804.34292.38 16552359

[5] Alves-Filho JC Spiller F Cunha FQ. Neutrophil paralysis in sepsis. Shock. (2010) 34 (Suppl. 1):15–21. 10.1097/SHK.0b013e3181e7e61b 20714263

[6] Qiao M Wang Y Xu X Lu J Dong Y Tao W. Mst1 is an interacting protein that mediates PHLPPs' induced apoptosis. Mol Cell. (2010) 38:512–23. 10.1016/j.molcel.2010.03.017 20513427

[7] Brognard J Newton AC. PHLiPPing the switch on Akt and protein kinase C signaling. Trends Endocrinol Metabol. (2008) 19:223–30. 10.1016/j.tem.2008.04.001 18511290 PMC2963565

[8] Warfel NA Newton AC. Pleckstrin homology domain leucine-rich repeat protein phosphatase (PHLPP): a new player in cell signaling. J Biol Chem. (2012) 287:3610–6. 10.1074/jbc.R111.318675 22144674 PMC3281723

[9] Li X Stevens PD Liu J Yang H Wang W Wang C. PHLPP is a negative regulator of RAF1, which reduces colorectal cancer cell motility and prevents tumor progression in mice. Gastroenterology. (2014) 146:1301–12.e1–10. 10.1053/j.gastro.2014.02.003 24530606 PMC3992173

[10] Arias E Koga H Diaz A Mocholi E Patel B Cuervo AM. Lysosomal mTORC2/PHLPP1/Akt regulate chaperone-mediated autophagy. Mol Cell. (2015) 59:270–84. 10.1016/j.molcel.2015.05.030 26118642 PMC4506737

[11] Baker B Geng S Chen K Diao N Yuan R Xu X. Alteration of lysosome fusion and low-grade inflammation mediated by super-low-dose endotoxin. J Biol Chem. (2015) 290:6670–8. 10.1074/jbc.M114.611442 25586187 PMC4358298

[12] Wen YA Li X Goretsky T Weiss HL Barrett TA Gao T. Loss of PHLPP protects against colitis by inhibiting intestinal epithelial cell apoptosis. Biochim Biophys Acta. (2015) 1852:2013–23. 10.1016/j.bbadis.2015.07.012 26187040 PMC4554835

[13] Rothschild DE Zhang Y Diao N Lee CK Chen K Caswell CC. Enhanced mucosal defense and reduced tumor burden in mice with the compromised negative regulator IRAK-M. EBioMed. (2017) 15:36–47. 10.1016/j.ebiom.2016.11.039 27939424 PMC5233813

[14] Diao N Zhang Y Chen K Yuan R Lee C Geng S. Deficiency in Toll-interacting protein (Tollip) skews inflamed yet incompetent innate leukocytes *in vivo* during DSS-induced septic colitis. Sci Rep. (2016) 6:34672. 10.1038/srep34672 27703259 PMC5050405

[15] Gallo RL Kim KJ Bernfield M Kozak CA Zanetti M Merluzzi L. Identification of CRAMP, a cathelin-related antimicrobial peptide expressed in the embryonic and adult mouse. J Biol Chem. (1997) 272:13088–93. 10.1074/jbc.272.20.13088 9148921

[16] Gobbetti T Coldewey SM Chen J McArthur S le Faouder P Cenac N. Nonredundant protective properties of FPR2/ALX in polymicrobial murine sepsis. Proc Natl Acad Sci USA. (2014) 111:18685–90. 10.1073/pnas.1410938111 25512512 PMC4284560

[17] Rios-Santos F Alves-Filho JC Souto FO Spiller F Freitas A Lotufo CM. Down-regulation of CXCR2 on neutrophils in severe sepsis is mediated by inducible nitric oxide synthase-derived nitric oxide. Am J Respir Crit Care Med. (2007) 175:490–7. 10.1164/rccm.200601-103OC 17138957

[18] Rothfuchs AG Trumstedt C Wigzell H Rottenberg ME. Intracellular bacterial infection-induced IFN-gamma is critically but not solely dependent on Toll-like receptor 4-myeloid differentiation factor 88-IFN-alpha beta-STAT1 signaling. J Immunol. (2004) 172:6345–53. 10.4049/jimmunol.172.10.6345 15128825

[19] Rajaram MV Butchar JP Parsa KV Cremer TJ Amer A Schlesinger LS. Akt and SHIP modulate Francisella escape from the phagosome and induction of the Fas-mediated death pathway. PLoS ONE. (2009) 4:e7919. 10.1371/journal.pone.0007919 19936232 PMC2775408

[20] Underhill DM Goodridge HS. Information processing during phagocytosis. Nat Rev Immunol. (2012) 12:492–502. 10.1038/nri3244 22699831 PMC5570470

[21] Chen J Tang H Hay N Xu J Ye RD. Akt isoforms differentially regulate neutrophil functions. Blood. (2010) 115:4237–46. 10.1182/blood-2009-11-255323 20332370 PMC2879106

[22] Piantadosi CA Suliman HB. Transcriptional control of mitochondrial biogenesis and its interface with inflammatory processes. Biochim Biophys Acta. (2012) 1820:532–41. 10.1016/j.bbagen.2012.01.003 22265687 PMC3307899

[23] Ran T Geng S Li L. Neutrophil programming dynamics and its disease relevance. Sci China Life Sci. (2017) 60:1168–77. 10.1007/s11427-017-9145-x 28971361

[24] Lee C Geng S Zhang Y Rahtes A Li L. Programming and memory dynamics of innate leukocytes during tissue homeostasis and inflammation. J Leukocyte Biol. (2017) 102:719–26. 10.1189/jlb.6MR0117-027RR 28476750 PMC5557635

[25] Yuan R Li L. Dynamic modulation of innate immunity programming and memory. Sci China Life Sci. (2016) 59:38–43. 10.1007/s11427-015-4998-x 26740103

[26] Morris MC Gilliam EA Li L. Innate immune programing by endotoxin and its pathological consequences. Front Immunol. (2014) 5:680. 10.3389/fimmu.2014.00680 25610440 PMC4285116

[27] Geng S Chen K Yuan R Peng L Maitra U Diao N. The persistence of low-grade inflammatory monocytes contributes to aggravated atherosclerosis. Nat Commun. (2016) 7:13436. 10.1038/ncomms13436 27824038 PMC5105176

[28] Yuan R Geng S Chen K Diao N Chu HW Li L. Low-grade inflammatory polarization of monocytes impairs wound healing. J Pathol. (2016) 238:571–83. 10.1002/path.4680 26690561 PMC4760849

[29] Futosi K Mocsai A. Tyrosine kinase signaling pathways in neutrophils. Immunol Rev. (2016) 273:121–39. 10.1111/imr.12455 27558332

[30] Heit B Tavener S Raharjo E Kubes P. An intracellular signaling hierarchy determines direction of migration in opposing chemotactic gradients. J Cell Biol. (2002) 159:91–102. 10.1083/jcb.200202114 12370241 PMC2173486

[31] Hawkins PT Stephens LR. PI3K signalling in inflammation. Biochim et Biophys Acta. (2015) 1851:882–97. 10.1016/j.bbalip.2014.12.006 25514767

[32] Grzechnik AT Newton AC. PHLPPing through history: a decade in the life of PHLPP phosphatases. Biochem Soc Trans. (2016) 44:1675–82. 10.1042/BST20160170 27913677 PMC5783572

[33] Reyes G Niederst M Cohen-Katsenelson K Stender JD Kunkel MT Chen M. Pleckstrin homology domain leucine-rich repeat protein phosphatases set the amplitude of receptor tyrosine kinase output. Proc Natl Acad Sci USA. (2014) 111:E3957–65. 10.1073/pnas.1404221111 25201979 PMC4183331

[34] Kovarik P Stoiber D Novy M Decker T. Stat1 combines signals derived from IFN-gamma and LPS receptors during macrophage activation. EMBO J. (1998) 17:3660–8. 10.1093/emboj/17.13.3660 9649436 PMC1170702

[35] Serezani CH Lewis C Jancar S Peters-Golden M. Leukotriene B4 amplifies NF-kappaB activation in mouse macrophages by reducing SOCS1 inhibition of MyD88 expression. J Clin Invest. (2011) 121:671–82. 10.1172/JCI43302 21206089 PMC3026722

[36] Pettitt D Arshad Z Smith J Stanic T Hollander G Brindley D. CAR-T Cells: a systematic review and mixed methods analysis of the clinical trial landscape. Mol Ther. (2018) 26:342–53. 10.1016/j.ymthe.2017.10.019 29248427 PMC5835018

