# Supplementary material for: Enhanced Neutrophil Immune Homeostasis Due to Deletion of PHLPP
Source: Front Immunol. 2019 Sep 6;10:2127. doi: 10.3389/fimmu.2019.02127 (PMC6742689; doi:10.3389/fimmu.2019.02127)
Supplement: Supplementary file 1 [file Data_Sheet_1.docx]

**Supplementary Figures and Legend**

**Enhanced Mucosal Immune homeostasis and Neutrophil Function due to Deletion of PHLPP**

Taojing Ran^1,#^, Yao Zhang^1 #^, Na Diao^1,#^, Shuo Geng^1^, Keqiang Chen^1^, Christina Lee^3^, Liwu Li^1^*

^1^Department of Biological Sciences, Virginia Tech, Blacksburg, VA24061; ^2^Translational Biology, Medicine, and Health Graduate Program, Virginia Tech

Keywords: Mucosal inflammation, Neutrophils, PHLPP, Immune dynamics, Colitis

Running Title: PHLPP and septic colitis

*Correspondence:

Liwu Li

970 Washington Street

Virginia Tech

Blacksburg, VA 24061-0910

Email: [lwli@vt.edu](mailto:lwli@vt.edu)


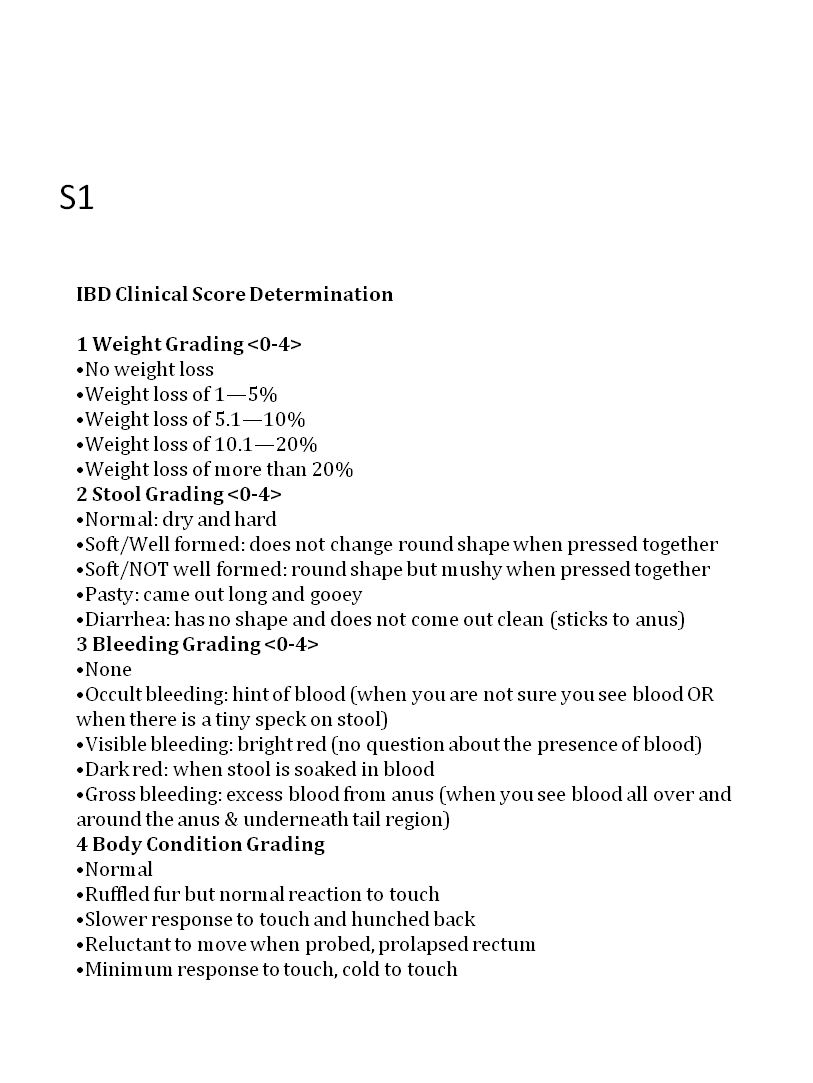


**Supplementary Figure S1.** The score system used to assess the clinical manifestation


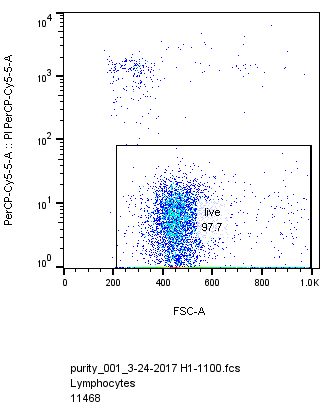

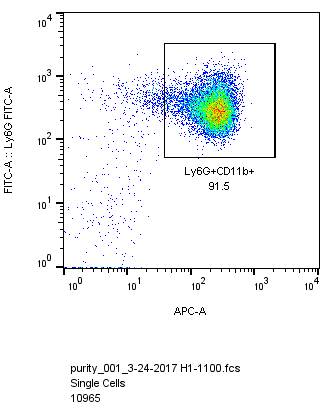


**FSC**

**PI**

**LY6G**

**CD11B**

**Supplementary Figure S2. Neutrophil purity measurement.** Representative flow plots of purified neutrophils from bone marrow.


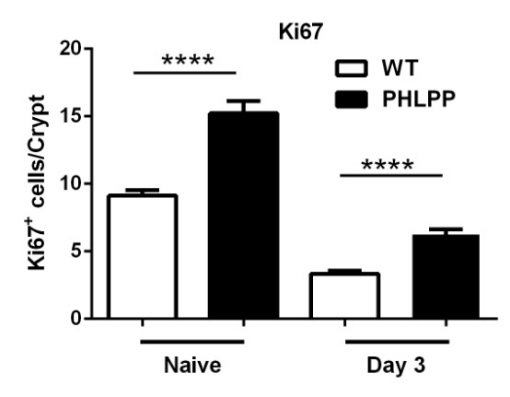


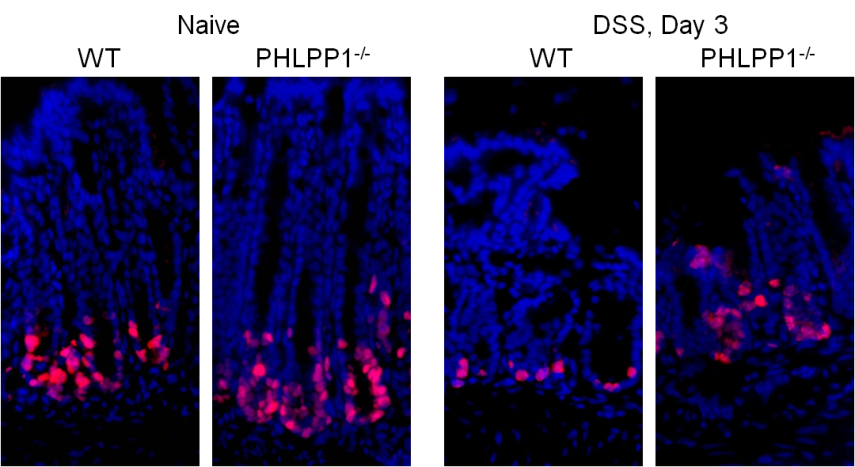


KI67

DAPI

WT

PHLPP

WT

PHLPP

Naïve

DSS, Day3

**Supplementary Figure S3. More proliferative cells in the colon crypt area were observed in PHLPP deficient mice.** Immunohistological staining of Ki67 (left) and analyses of cell count/crypt (right). Scar bar represents 50 μm. Data were represented as mean ± SEM, ***p < 0.001.


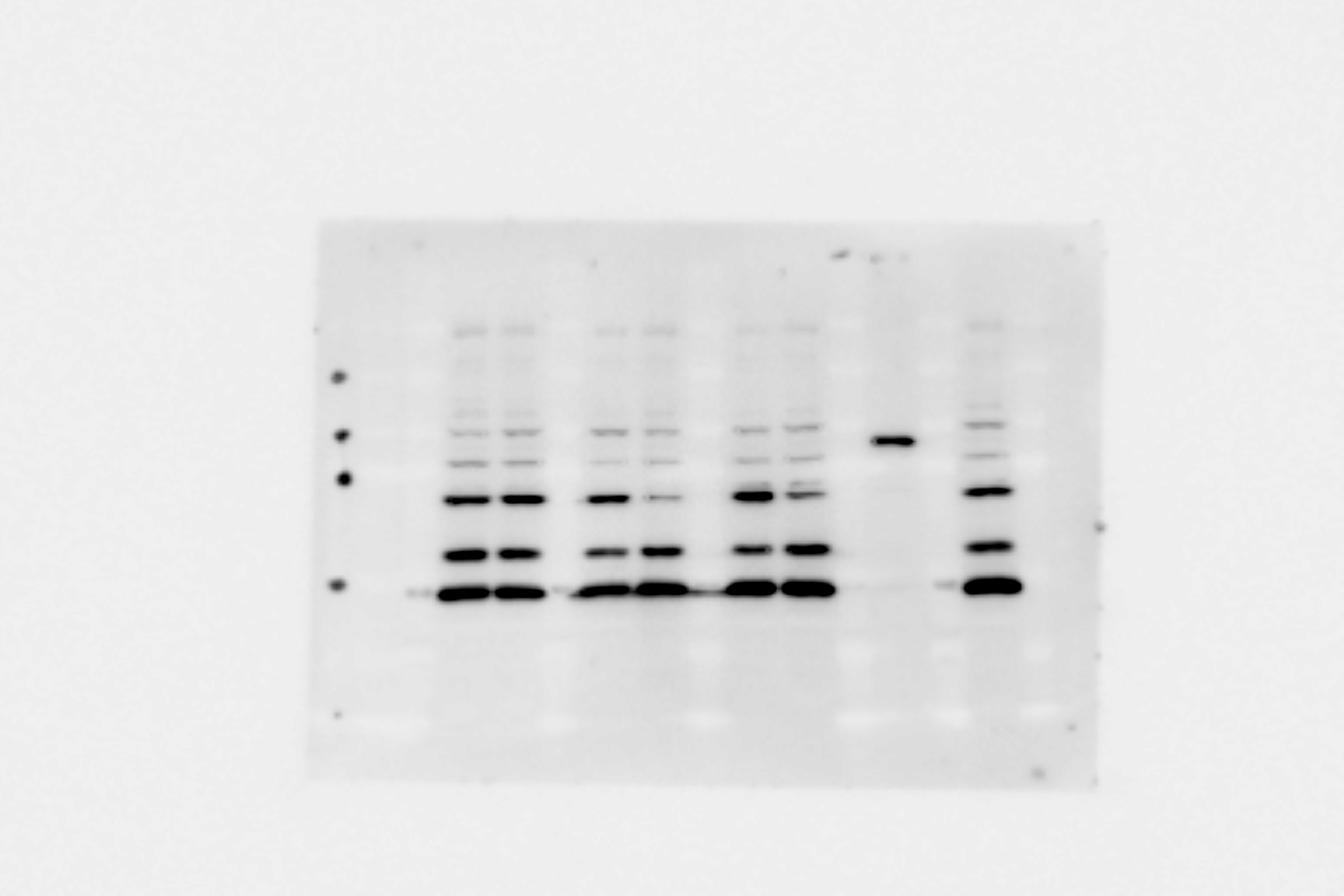


**anti-PHLPP**

**DSS, Day3**

**Colon**

**anti-β-ACTIN**


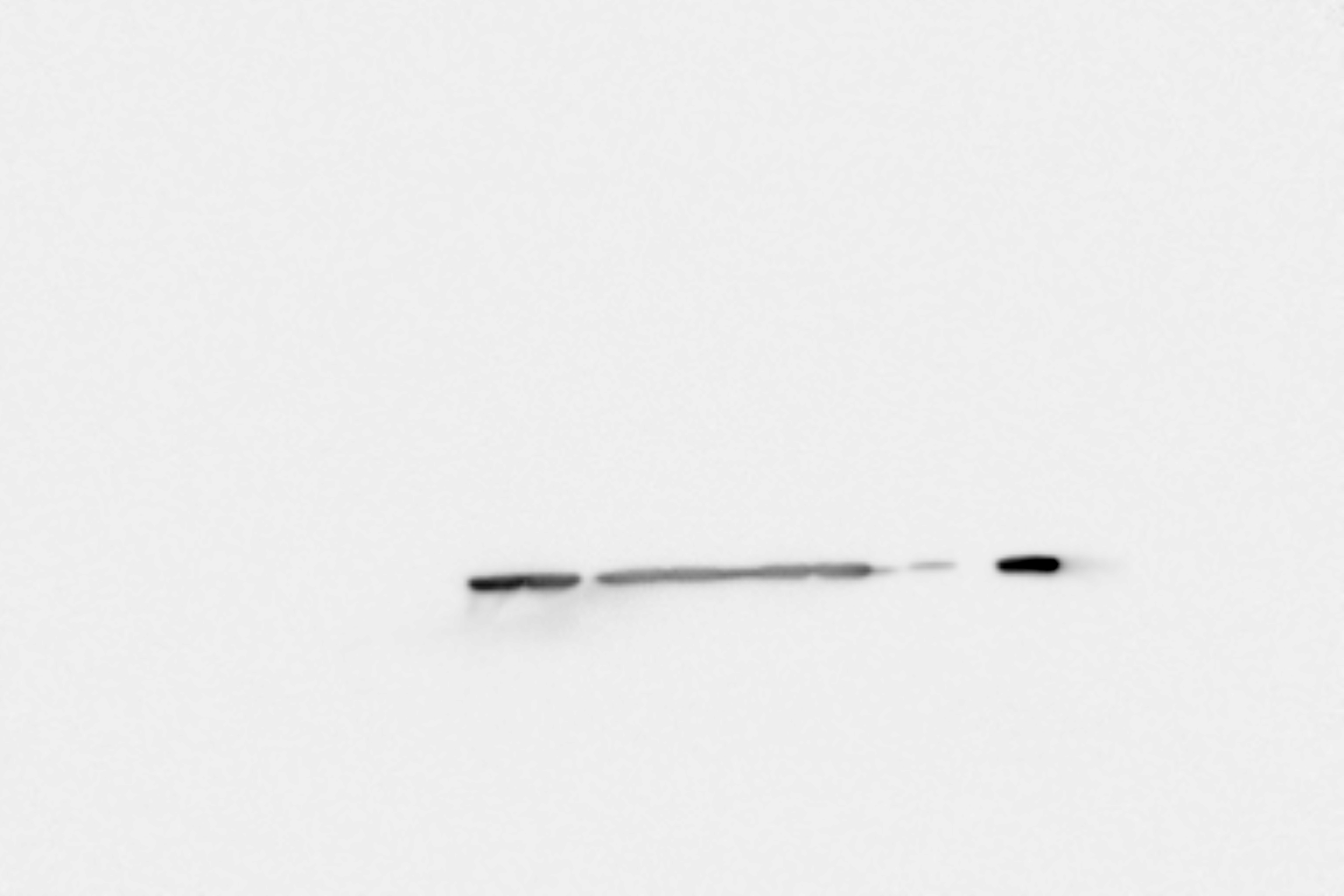


**Supplementary Figure S4. Examination of PHLPP expression in neutrophils *in vivo*.** Immunoblotting of neutrophil lysate. The neutrophils were isolated from colons after mice were fed with DSS for 3 days.


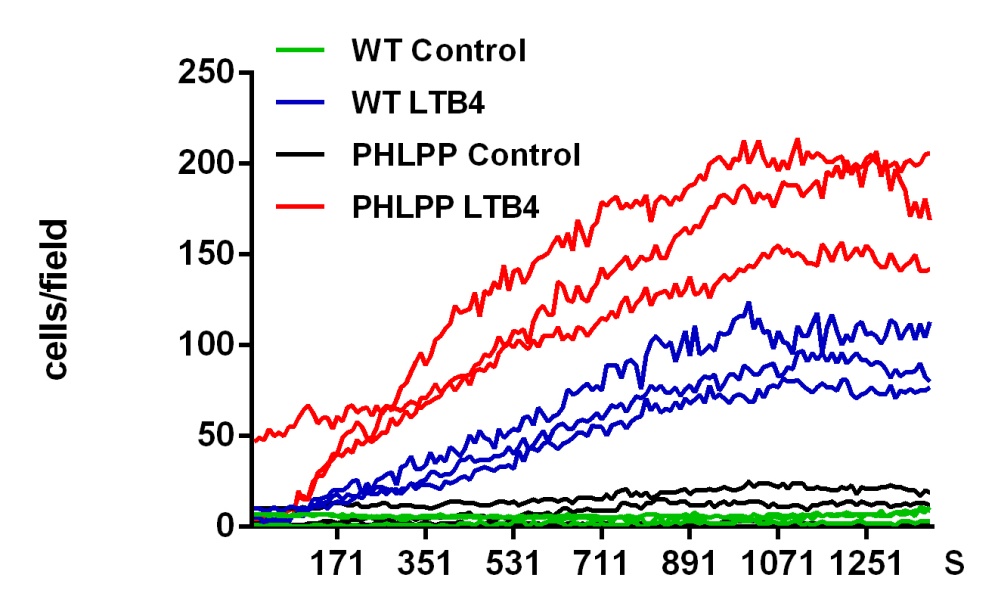


**Supplementary Figure S5. PHLPP deficiency enhanced migratory potential toward LTB4.** Dynamic migration ability of neutrophil via microfluidic assay for neutrophil migration towards LTB4 (100nM).
